# The Impact of Bilateral Salpingectomy on Ovarian Reserve: A Prospective Analysis of Hormonal, Ultrasound and Clinical Correlations

**DOI:** 10.3390/medsci14030416

**Published:** 2026-07-22

**Authors:** Teodora Radu, Matyas Mar, Marius Gliga, Claudiu Marginean

**Affiliations:** 1 Doctoral School of Medicine, George Emil Palade University of Medicine, Pharmacy, Sciences and Technology of Targu Mures, Gheorghe Marinescu Street No. 38, 540142 Targu Mures, Romania; 2 Department of Obstetrics and Gynecology 2, George Emil Palade University of Medicine, Pharmacy, Sciences and Technology of Targu Mures, Gheorghe Marinescu Street No. 38, 540136 Targu Mures, Romania

**Keywords:** bilateral salpingectomy, ovarian reserve, AMH, FSH, AFC, WHQ

## Abstract

**Background**: Opportunistic salpingectomy is widely used in gynecological surgery as a preventive measure against ovarian carcinoma. Questions have been raised about the potential impact of salpingectomy on ovarian function, given the possibility of ovarian vascularity disruption through the utero-ovarian arch located in the mesosalpinx. The aim of this study was to evaluate the short-term effects of bilateral salpingectomy on ovarian reserve. **Methods**: This prospective cohort study included forty premenopausal women, aged 34–50 years, who underwent hysterectomy with bilateral salpingectomy and ovarian preservation for benign uterine conditions. Ovarian reserve was evaluated before surgery and three months postoperatively, using serum anti-Müllerian hormone (AMH), follicle-stimulating hormone (FSH) and antral follicle count (AFC). Health-related quality of life was assessed using the Women’s Health Questionnaire (WHQ). **Results**: Serum FSH levels increased significantly after surgery, while AMH levels decreased significantly (both *p* < 0.05). No significant postoperative changes were observed in AFC. The overall WHQ score increased significantly, indicating a mild deterioration in health-related quality of life. The greatest postoperative changes were observed in the depressive, somatic and sexual domains, where menstrual symptoms improved following surgery. No significant correlation was found between WHQ scores and hormonal or ultrasonographic markers of ovarian reserve. **Conclusions**: Bilateral salpingectomy performed during hysterectomy was associated with significant short-term hormonal changes, while AFC remained stable. Although patient-reported quality of life slightly worsened after surgery, these changes were not correlated with hormonal or ultrasonographic markers of ovarian reserve. Larger controlled studies with longer follow-up are required to determine the long-term clinical significance of these findings.

## 1. Introduction

Due to its asymptomatic course and its late-stage diagnosis, ovarian cancer remains a major cause of gynecological cancer-related death, underscoring the imperative need for early detection, and most importantly, for implementing effective primary prevention strategies [1].

Salpingectomy is defined as the surgical excision of one or both fallopian tubes, most commonly indicated for permanent sterilization or for tubal disorder management, such as ectopic pregnancy or hydro/pyo-salpinx. In contrast, opportunistic salpingectomy involves the removal of both fallopian tubes—macroscopically normal—during ovary-sparing pelvic surgeries [2]. Opportunistic salpingectomy has become a widely adopted procedure in gynecological surgery and is considered a preventive strategy for ovarian carcinoma, supported by growing evidence indicating that certain ovarian cancer subtypes originate from the tubal epithelium. This concept was promoted by a group of gynecologic oncologists in British Columbia and Canada in 2010, with the aim of educating gynecologic practitioners about the potential preventive role of bilateral salpingectomy in ovarian carcinoma development [3,4,5,6]. They also advocated for replacing tubal ligation with bilateral salpingectomy and performing opportunistic salpingectomy at the time of ovarian-sparing hysterectomy in low-risk patients [7,8,9]. In contrast, for high-risk patients carrying a germline mutation, like BRCA 1 or BRCA 2, risk-reducing salpingo-oophorectomy is recommended [9].

Salpingectomy proved to be a safe and feasible alternative to tubal ligation when performed for sterilization in the post-partum period, during cesarean section or as an interval procedure. Current evidence suggests that, although it may be associated with a prolonged operative time, it does not significantly increase intraoperative or postoperative complications [10].

However, the widespread implementation of this procedure raises questions about the consequences on ovarian homeostasis, as the utero-ovarian vascular arch crosses the mesosalpinx and could be compromised during surgical dissection. Furthermore, some studies suggest differing ovarian function impairment post-salpingectomy, depending on the type of approach and whether electrosurgery is used. Ovarian tissue damage may arise, whether from the disruption of the collateral vascular supply, direct parenchymal injury or lateral thermal dispersion from energy devices. Beyond the impact on fertility, altered ovarian function may have consequences on women’s cardiovascular health, bone density, and cognitive function. Even subtle alterations in ovarian perfusion may theoretically affect follicular development and ovarian reserve; however, current clinical evidence regarding this association remains inconsistent [11,12].

In the context of the expanded indications for performing prophylactic salpingectomy, it is primordial to balance the benefit of the intervention and the potential negative impact on ovarian function with the repercussions of menopause onset on all systems: cardiovascular, nervous, skeletal, digestive, excretory, genital, and others.

Serum follicle-stimulating hormone (FSH) levels are widely used as an indirect marker of declining ovarian function, with elevated concentrations suggesting diminished follicular feedback and progression toward menopause. In parallel, anti-Müllerian hormone (AMH), secreted by granulosa cells of preantral and small antral follicles, is considered one of the most reliable markers of ovarian reserve, due to its cycle stability and strong correlation with the remaining follicular pool [13]. Ultrasonographic analysis of the antral follicle count (AFC) constitutes a further complementary evaluation, providing a direct estimate of ovarian follicular reserve. At the same time, FSH, AMH, and AFC represent key parameters for evaluating reproductive endocrine status and the potential impact of gynecological surgery on ovarian function [14].

The Women’s Health Questionnaire (WHQ) is a validated instrument used for assessing women’s health-related quality of life, including physical and psycho-emotional symptoms commonly associated with menopausal manifestations. Its broad clinical applicability makes it useful for evaluating outcomes following gynecologic surgery [15].

Therefore, the present prospective study aimed to evaluate the short-term impact of bilateral salpingectomy performed during hysterectomy on ovarian reserve, using hormonal (AMH and FSH), ultrasonographic (AFC), and patient-reported (WHQ) outcome measures.

## 2. Materials and Methods

### 2.1. Study Design and Patient Selection

This study was designed as a prospective, observational pre- and post-intervention cohort study, and was conducted between February 2020 and February 2021. Ethical approval was obtained from the Ethics Review Board of the “George Emil Palade” University of Medicine, Pharmacy, Science, and Technology of Targu-Mures, Mures County, Romania (No. 741/18.02.2020) 18 February 2020. Written informed consent was obtained from all participants prior to enrollment. All procedures performed in this study were conducted in accordance with the ethical principles of the Declaration of Helsinki and its subsequent amendments.

A total of 40 premenopausal women aged 34–50 years who underwent hysterectomy with bilateral salpingectomy between February 2020 and February 2021, during which both ovaries were preserved for benign uterine conditions, were included in the study.

The inclusion criteria included the following: age within the specified range, both ovaries preserved with no macroscopic ovarian pathology, a valid indication for hysterectomy via abdominal or laparoscopic approach, and documented informed consent for participation. Exclusion criteria comprised the following: underlying ovarian pathology, menopausal status, previous adnexal surgery, suspected or confirmed malignancy and pre-existing endocrine disorders or the use of hormonal therapy within the previous three months. This stratification of cases contributed to sample homogeneity and reduced the influence of confounding variables.

Menopausal symptoms and health-related quality of life were assessed using the Women’s Health Questionnaire (WHQ), administered preoperatively and 3 months after surgery. In parallel, ovarian function was evaluated before and 90 days following the intervention by measuring serum FSH and AMH levels and determining the antral follicle count (AFC).

### 2.2. Study Objectives

The primary objective of the study was to investigate whether bilateral salpingectomy performed during hysterectomy for benign uterine pathologies adversely affects ovarian function in premenopausal women with ovarian preservation.

Secondary objectives included assessing postoperative changes in ovarian reserve and investigating their potential association with menopausal symptoms and health-related quality of life.

The study followed three directions. Firstly, clinical outcomes were assessed using the Women’s Health Questionnaire (WHQ), a validated instrument designed to evaluate menopausal symptoms and health-related quality of life, allowing the identification of potential postoperative changes in patient-reported outcomes.

Secondly, ovarian reserve was evaluated through the measurement of serum anti-Müllerian (AMH) and follicle-stimulating hormone (FSH) levels, reflecting ovarian function and reproductive potential following bilateral salpingectomy.

Thirdly, using three-dimensional (3D) ultrasound, ultrasonographic assessment of the antral follicle count (AFC) was performed to further analyze ovarian reserve and corroborate potential postoperative changes in ovarian function.

Finally, correlations between clinical symptomatology, hormonal and ultrasonographic parameters were explored.

### 2.3. Assessment Protocol

#### 2.3.1. Assessment of Menopausal Symptoms (WHQ)

The presence of menopausal symptoms and their impact on the quality of life were assessed using the Women’s Health Questionnaire (WHQ), licensed for use through the Mapi Research Trust (MRT), authorized for the Romanian-translated version (license number: 130305). This instrument helped us in evaluating domains, such as depression and anxiety, somatic symptoms, memory, vasomotor symptoms, sexual behavior, sleep problems, menstrual symptoms, and attractiveness. The questionnaire was administered preoperatively and repeated 3 months postoperatively. This approach helped in evaluating the impact of surgery on patients’ quality of life and on short-term ovarian function. Furthermore, we aimed to investigate whether there was any correlation between hormonal levels, antral follicle count and clinical presentation.

#### 2.3.2. Hormonal Assessment

Serum anti-Müllerian hormone (AMH) levels were measured using an enzyme-linked immunosorbent assay (ELISA) kit (EIA-6053;DRG Instruments GmbH, Marburg, Germany), following the manufacturer’s instructions. Blood samples were centrifuged 30 min after collection. The resulting serum was separated and stored at −20 °C until analysis. Before processing the probe, the samples were brought to room temperature. Absorbance was measured at 620 nm using a fully automated DSX ELISA analyzer (DYNEX Technologies, Chantilly, VA, USA). Defined as two standard deviations above the mean absorbance of 20 replicate measurements of the zero calibrator, assay sensitivity was approximately 0.02 ng/mL. The limitations of serum AMH testing include biological variability, the influence of sample handling and storage conditions. In contrast with serum FSH, it is a relatively stable hormone, independent of menstrual cycle phase.

Serum follicle-stimulating hormone (FSH) was assessed using a kit named DRG FSH ELISA (EIA-1288; DRG Instruments GmbH, Marburg, Germany), based on the sandwich principle. The same methods of blood collection, centrifugation and storage used for AMH were applied. The absorbance was determined at 450 nm using the fully automated DSX ELISA analyzer (DYNEX Techologies, Chantilly, VA, USA). The results have been calculated automatically using a 4-parameter curve fit. The analytical sensitivity was calculated from the mean plus two standard deviations of 20 replicate analyses of standard 0 and was found to be 0.856 mIU/mL. The limitations in testing serum FSH include cycle-dependent variability, biological and inter-individual variability and the decline in late markers of ovarian function.

#### 2.3.3. Ultrasonographic Assessment

**Figure 1:**
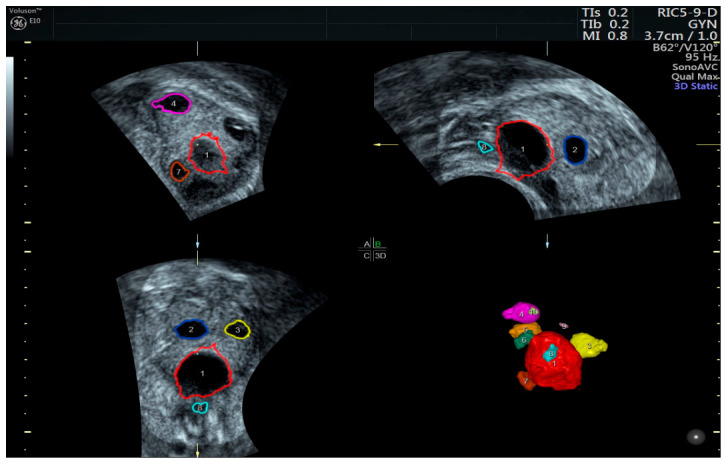
Representative three-dimensional ovarian ultrasound image obtained using the integrated SonoAVC™ (Sonography-based Automated Volume Count) software. (A) Plane A of the multiplanar display showing automatically detected antral follicles. (B) Plane B of the multiplanar display. (C) Plane C of the multiplanar display. (3D) Three-dimensional rendered reconstruction of the segmented follicles generated by SonoAVC. Different colors identify individual follicles automatically recognized by the software.

Antral follicle count (AFC) was assessed using a high-frequency volumetric vaginal probe (5–9 MHz) in accordance with current international recommendations. Both ovaries were examined with three-dimensional (3D) volume acquisitions. Follicle identification and automated counting were performed using the integrated SonoAVC™ (Sonography-based Automated Volume Count), software (BT20) on a Voluson™ E10 ultrasound system (GE Healthcare Austria GmbH & Co OG, Zipf, Austria), that provides a more accurate and reproducible assessment of the antral follicles than manual counting (Figure 1). Each follicle was assigned a different color, facilitating visualization and counting. Total AFC was calculated as the sum of the antral follicles identified in both ovaries. All ultrasound examinations were performed by the same experienced operator to minimize interobserver variability.

### 2.4. Surgical Technique and Intraoperative Protective Measures

The surgical procedure followed internal protocols for performing hysterectomy with bilateral salpingectomy, with the patient consenting to the surgical intervention and taking part in the study and being informed of the associated risks. Preoperatively electrocardiogram and chest X-ray were performed. Prophylactic antibiotherapy was systematically conducted, a bladder catheter was installed, routine preoperative blood tests were conducted (hemogram, coagulogram, renal and liver function, blood type with rhesus factor, and urine analysis) and vaginal cultures and Pap smears were carried out.

Hysterectomy with opportunistic salpingectomy was performed via laparotomy or laparoscopy, depending on several factors, such as uterine size, underlying pathology, prior abdominal or pelvic surgery, the presence of adhesions and surgeon expertise. The strategy for preserving ovarian function included intraoperative assessment of ovarian tissue viability, minimizing the mechanical trauma of the ovaries; judicious use of electrosurgery energy was applied in order to reduce collateral thermal spread and limit the distant effect of tissue heating. Whenever technically feasible, excessive bipolar coagulation close to the ovarian hilum was avoided in an attempt to preserve ovarian vascularization.

### 2.5. Statistical Analysis

Data analysis was performed in IBM SPSS Statistics version 26.0 (IBM Corp., Armonk, NY, USA). Continuous variables were expressed as mean ± standard deviation (SD) for normally distributed data or as median and interquartile range (IQR) when normality assumptions were not met. The distribution of continuous variables was assessed using the Shapiro–Wilk test.

Pre- and postoperative measurements were compared using the paired-samples t-test for normally distributed variables and the Wilcoxon signed-rank test for non-normally distributed variables. Correlations between ovarian reserve markers and Women’s Health Questionnaire (WHQ) scores were evaluated using Spearman’s rank correlation coefficient. To improve the interpretation of statistical significance, effect sizes and 95% confidence intervals were reported for parametric analyses where appropriate. Statistical significance was defined as a two-sided *p* value < 0.05.

## 3. Results

### 3.1. Baseline Characteristics of the Study Population

A total of 40 premenopausal women were included in the final analysis. The mean age of the study population was 43.8 years (range, 34–50 years). All participants completed both the preoperative and the 3-month postoperative assessment. The baseline demographic and surgical characteristics of the study population are summarized in Table 1.

### 3.2. Changes in Serum FSH Levels

Comparative analysis using the paired-samples t-test demonstrated a statistically significant increase in serum FSH concentrations, from 9.21 ± 7.69 mIU/mL preoperatively to 12.06 ± 8.95 mIU/mL postoperatively, with a mean difference of + 2.8 mIU/mL (95% CI: 1.01–4.69). This increase was statistically significant (t(39) = 3.13, *p* = 0.0034) and associated with a moderate effect size (Cohen’s dz = 0.49).

**Figure 2:**
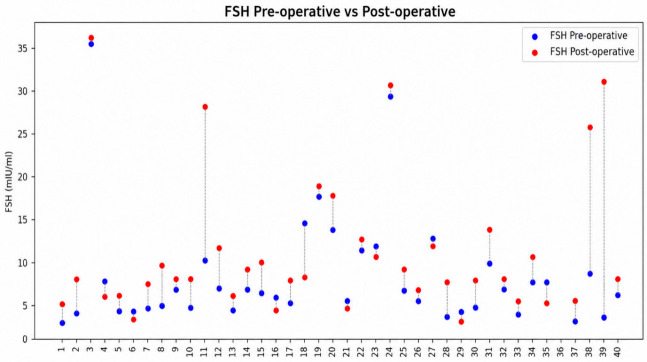
Individual pre- and postoperative FSH values.

The data represented in Figure 2 indicates FSH values for each patient, comparing preoperative and 3 months postoperative measurements, highlighting the specific changes for each case.

### 3.3. Changes in Ovarian Reserve Parameters Following Bilateral Salpingectomy

Serum anti-Müllerian hormone levels (AMH) were collected both before hysterectomy with bilateral salpingectomy and three months after the intervention. Given the asymmetric distribution of AMH values, the Wilcoxon signed-rank test was chosen.

The test results revealed the following:

- Test statistic = 118.0;
- *p*-value = 3.3 × 10^−5^.

The null hypothesis (H0)—according to which pre- and postoperative AMH levels do not differ significantly—was rejected in favor of the alternative (H_1_), which assumes a decrease in AMH after salpingectomy. The postoperative decrease in serum AMH was statistically significant (Wilcoxon signed-rank test, *p* = 3.3 × 10^−5^).

The Wilcoxon signed-rank test did not demonstrate a statistically significant postoperative change in AFC (*p* = 0.12).

The relationships between variables were assessed using Spearman’s coefficient, which is appropriate for non-parametric data. Significant positive correlations were identified between the evaluated parameters, with AFC showing the highest correlation coefficient, followed by AMH and FSH (Table 2). According to Spearman’s rank correlation analysis, the observed correlation was highly statistically significant (*p* < 0.001).

As shown in Table 3, the correlation pattern among ovarian reserve parameters remained largely unchanged following surgery. Moderate negative correlations were observed between FSH and AMH, as well as between FSH and AFC, both pre- and postoperatively. In contrast, a strong positive correlation between AMH and AFC was maintained at both assessment time points.

### 3.4. Changes in Women’s Health Questionnaire (WHQ) Scores

**Figure 3:**
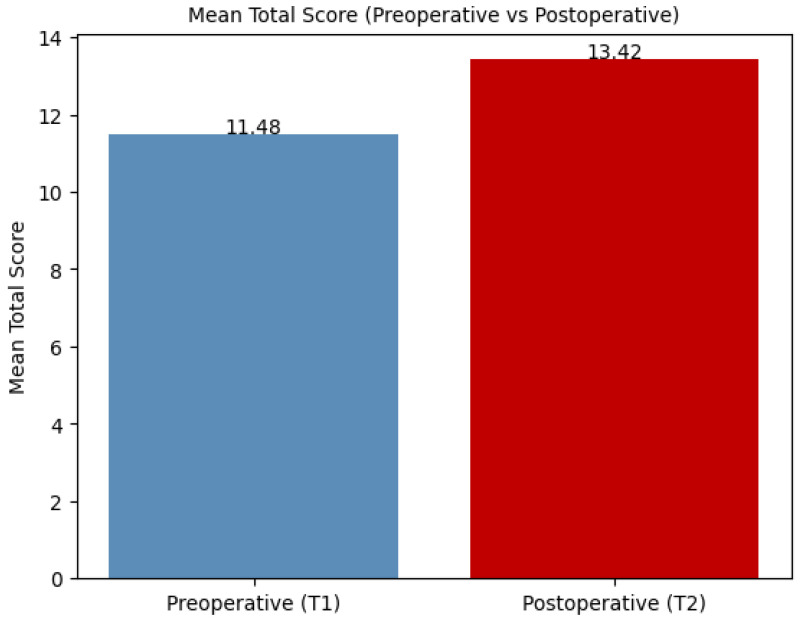
The impact of the surgical intervention. T1—preoperative; T2—postoperative.

As shown in Figure 3, a statistically significant increase in the total WHQ was observed following surgery, from 11.48 at baseline (T1) to 13.42 at postoperative assessment (T2), with *p* < 0.001, reflecting a higher burden of reported symptoms after hysterectomy with bilateral salpingectomy.

**Figure 4:**
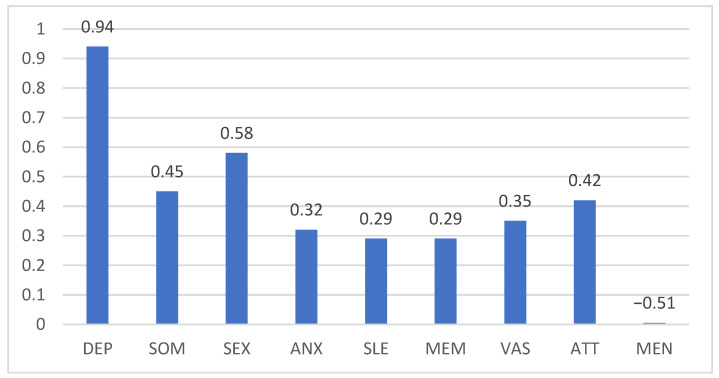
Mean difference (T2-T1) across domains (DEP—depressive symptoms, SOM—somatic symptoms, MEM—memory, VAS—vasomotor symptoms, ANX—anxiety, SEX—sexual symptoms, SLE—sleep disturbances, MEN—menstrual symptoms, ATT—attractiveness).

Figure 4 presents the mean postoperative changes across the individual WHQ domains. The largest mean changes were observed in DEP, SEX and SOM domains, whereas the MEM, VAS and SLE domains showed only minor changes. In contrast, the MEN (menstrual symptoms) domain demonstrated the greatest improvement following surgery, with a mean score reduction of 1.51 points. The individual domain changes shown in Figure 4 are presented descriptively.

### 3.5. Correlation Between Total WHQ Scores and Hormonal Parameters

As illustrated in Table 4, Correlation analysis between total WHQ and the evaluated variables revealed no statistically significant associations at either T1 or T2, with all variables demonstrating weak correlation coefficients and non-significant *p*-values (*p* > 0.05).

## 4. Discussion

### 4.1. Interpretation of the Findings

Using a comprehensive assessment that included hormonal, ultrasonographic and patient-reported outcome measures, this prospective study evaluated the short-term impact of bilateral salpingectomy performed during hysterectomy with ovarian preservation on ovarian reserve. The main findings indicated significant short-term hormonal changes characterized by a postoperative increase in FSH and a decrease in AMH, whereas AFC remained stable throughout the follow-up period. A significant increase in overall WHQ scores was observed, indicating a higher postoperative symptom burden. Although significant postoperative hormonal changes were observed, these changes were not accompanied by significant alterations in AFC or by evidence of clinically meaningful short-term ovarian dysfunction. Therefore, the statistical significance of these endocrine changes should not be interpreted as equivalent to immediate clinical significance.

Although a statistically significant increase in FSH levels was observed, postoperative values remained within the physiological range and were therefore unlikely to indicate clinically relevant short-term ovarian function impairment. The substantial interindividual variability in FSH levels—with some patients presenting marked postoperative increases and others showing minimal or no change—highlights the importance of a personalized approach in postoperative monitoring. This finding is consistent with previous reports suggesting that, although bilateral salpingectomy may induce subtle changes in ovarian reserve markers, these variations generally remain within the physiological range and are not associated with clinical ovarian insufficiency [12].

Furthermore, the absence of a statistically significant postoperative change in AFC, despite significant hormonal alterations, suggests that hormonal and ultrasonographic markers may reflect different aspects of ovarian reserve during the early postoperative period. These findings support a complementary interpretation of AMH and AFC when assessing ovarian reserve, as each marker provides different but complementary information regarding ovarian reserve. This complementary approach is consistent with current evidence indicating that AMH and AFC reflect different biological aspects of ovarian reserve and should therefore be interpreted together, rather than as interchangeable markers of ovarian function [16]. AMH is considered one of the most sensitive biomarkers of ovarian reserve because it reflects the pool of small growing follicles, whereas AFC provides a direct ultrasonographic estimate of the antral follicle population. Although these markers are strongly correlated, they may respond differently following surgical intervention, reflecting distinct biological aspects of ovarian function rather than identical physiological processes. Isolated short-term hormonal changes should therefore be interpreted cautiously and in conjunction with ultrasonographic findings and the clinical context [16,17]. The substantial interindividual variability observed in both hormonal and ultrasonographic parameters, reflected by the large standard deviations, further supports heterogeneous ovarian response following surgery. A substantial decline in AMH levels was also observed by Tavana et al. six months after bilateral salpingectomy, with a more pronounced reduction in patients undergoing abdominal surgery compared to those treated laparoscopically [18]. Likewise, in a randomized controlled trial, Vignarajan et al. demonstrated a significant decrease in both AMH and AFC levels (*p* < 0.001) after bilateral salpingectomy [19], findings that were further supported by additional studies evaluating the effects of salpingectomy on ovarian reserve [20,21]. Although AMH is regarded as one of the most sensitive biomarkers of ovarian reserve, it primarily reflects the activity of small growing follicles rather than the entire primordial follicle pool. Consequently, isolated short-term hormonal changes should not be automatically interpreted as evidence of clinically significant ovarian dysfunction, particularly in the absence of concordant ultrasonographic findings or clinical manifestations [18].

Our results did not demonstrate a statistically significant change in AFC (*p* = 0.12). Similar ultrasonographic stability has also been reported by Venturella et al. (2017), Rustamov et al. (2016), Lashin et al. (2021) and Wang et al. (2021), all of whom described minimal or no postoperative variation in AFC [22,23,24,25]. Taken together, these findings suggest that hormonal and ultrasonographic markers should be interpreted together rather than individually when assessing the short-term effects of opportunistic salpingectomy on ovarian reserve.

Correlation analysis indicated a stable relationship between ovarian reserve parameters before and after intervention. The expected inverse correlations between FSH and both AMH and AFC, together with the positive correlation between AMH and AFC, are consistent with reproductive physiology and support the complementary role of these markers in assessing ovarian reserve. In contrast to these relatively stable relationships among ovarian reserve markers, the increase in WHQ scores indicates worsening symptomatology following surgery. It is worth emphasizing that hormonal parameters did not correlate with symptom severity, suggesting that postoperative physical and emotional changes are not directly related to altered ovarian function. This finding is consistent with previous reports indicating that menopausal symptoms may evolve independently of endocrine changes, and that postoperative recovery represents a complex process that significantly influences psychosomatic balance [26,27,28,29,30].

From a clinical perspective, these results emphasize the importance of preoperative counseling and individualized postoperative follow-up. While ovarian reserve appears to be preserved, the increase in symptom burden highlights the need to assess patient-reported outcomes and quality of life, especially in premenopausal women.

Several studies and meta-analyses have concluded that opportunistic salpingectomy does not impair ovarian reserve or ovarian vascularization, encouraging its use as a means to prevent ovarian cancer [31,32,33,34,35,36,37]. In this context, our study is consistent with the existing literature, suggesting that even if subtle changes in ovarian reserve markers may appear, they generally remain within physiological limits and are not associated with ovarian failure.

The biological mechanisms underlying the observed endocrine changes remain incompletely understood. Recent experimental evidence suggests that ovarian reserve is regulated not only by vascular integrity, but also by complex molecular pathways involved in follicular survival, oxidative stress, inflammation, and granulosa cell function [38,39]. These mechanistic findings provide a plausible biological framework for the subtle endocrine alterations observed in the present study, although direct clinical evidence remains limited.

### 4.2. Study Strengths and Limitations

Despite the relatively modest sample size, the present study has several important strengths. Its prospective design, standardized surgical technique, and complete follow-up of all participants strengthen the internal validity of the findings. Furthermore, ovarian function was investigated using a multidimensional approach that included hormonal measurements (AMH, FSH), ultrasonographic assessment (AFC) and patient-reported outcomes through the Women’s Health Questionnaire (WHQ). According to the available literature, few studies have simultaneously evaluated these complementary parameters in women undergoing bilateral salpingectomy, and the inclusion of WHQ provides additional insight into the subjective impact of surgery, which remains insufficiently explored in the current literature.

Nevertheless, several limitations should be acknowledged when interpreting the current findings. Firstly, the relatively small sample size (*n* = 40) may have limited the statistical power to detect subtle differences, particularly in subgroup analyses, and may reduce the generalizability of the results. Secondly, the follow-up period was limited to three months and therefore reflects only the early postoperative effects of bilateral salpingectomy. Longer follow-up is required to determine whether the observed hormonal changes persist, resolve, or progress over time. Thirdly, the absence of a control group undergoing hysterectomy alone limits the interpretation of the findings, as the observed endocrine changes may reflect the combined effects of hysterectomy and bilateral salpingectomy rather than salpingectomy alone. Moreover, the physiological stress associated with major surgery may transiently influence the hypothalamic–pituitary–ovarian axis through postoperative changes in stress-related hormones, such as cortisol and prolactin, potentially contributing to the observed postoperative increase in FSH. In addition, postoperative pain, reduced mobility, and the recovery process, particularly following laparotomy, may have temporarily influenced patient-reported outcomes and should therefore be considered when interpreting the WHQ results. Finally, the inclusion of women up to 50 years of age may represent an additional source of biological variability, as some participants were approaching the menopausal transition, during which physiological changes in ovarian reserve markers may occur independently of surgery.

Despite these limitations, the prospective design, complete follow-up, and comprehensive assessment of ovarian reserve provide valuable prospective clinical data regarding the early effects of opportunistic bilateral salpingectomy. Future multicenter prospective studies including larger cohorts, appropriate control groups, longer follow-up periods, and additional markers of ovarian perfusion and ovarian function are warranted to further clarify the long-term impact of this procedure on ovarian reserve and patient well-being.

## 5. Conclusions

In conclusion, bilateral salpingectomy performed during hysterectomy for benign uterine disease was associated with significant short-term hormonal changes, including a postoperative increase in FSH and a decrease in AMH, whereas AFC remained stable. Importantly, although FSH increased significantly, postoperative values remained within the physiological range, and no evidence of clinically meaningful short-term ovarian function impairment was identified.

Following hysterectomy, a significant postoperative increase in symptom burden was observed, particularly in the psycho-emotional, somatic, and sexual domains, whereas menstrual symptoms improved. No significant correlations were identified between WHQ scores and ovarian reserve markers, suggesting that patient-reported postoperative symptoms may not directly reflect endocrine or ultrasonographic changes in ovarian reserve.

Overall, these findings suggest that bilateral salpingectomy with ovarian preservation appears to be safe in the short term, while emphasizing the importance of a comprehensive postoperative assessment integrating both biological markers and patient-reported outcomes.

Further multicenter prospective studies including larger cohorts, appropriate control groups, and longer follow-up are needed to clarify the long-term impact of opportunistic salpingectomy on ovarian reserve, endocrine function, and patient well-being.

## Figures and Tables

**Table 1:** Baseline demographic and surgical characteristics of the study population.

Characteristic	Value
Number of patients	40
Age, years	43.8 (range 34–50)
Total hysterectomy	32 (80.0%)
Subtotal hysterectomy	8 (20.0%)
Laparoscopy	17 (42.5%)
Laparotomy	23 (57.5%)
Preoperative, AMH—ng/mL	9.62 ± 8.02
Preoperative, FSH—mIU/mL	9.21 ± 7.69
Preoperative, AFC	9.83 ± 3.26

**Table 2:** Synthesis of central values and dispersion of the investigated parameters and longitudinal correlations. FSH = follicle-stimulating hormone; AMH = anti-Müllerian hormone; AFC = antral follicle count.

Parameter	Preoperative Mean Value and Standard Deviation	Postoperative Mean Value and Standard Deviation	Spearman Coefficient
FSH (mIU/mL)	9.21 ± 7.69	12.06 ± 8.95	0.76
AMH (ng/mL)	9.62 ± 8.02	7.32 ± 6.87	0.82
AFC	9.83 ± 3.26	9.23 ± 3.52	0.85

**Table 3:** Pre- and postoperative correlations between ovarian reserve parameters. FSH = follicle-stimulating hormone; AMH = anti-Müllerian hormone; AFC = antral follicle count.

Variables	Preoperative (r)	Postoperative (r)
FSH–AMH	−0.45	−0.42
AMH–AFC	0.76	0.74
FSH–AFC	−0.40	−0.38

**Table 4:** Correlation between total WHQ scores (pre- and postoperatively) and hormonal parameters FSH, AMH and AFC. FSH—follicle stimulating hormone; AMH—anti-Müllerian hormone; AFC—antral follicle count; T1—preoperatively; T2—postoperatively.

Variables	Correlation T1	*p*-Value T1	Correlation T2	*p*-Value T2
FSH	−0.15	0.22 (ns)	−0.18	0.15 (ns)
AMH	+0.10	0.35 (ns)	+0.12	0.29 (ns)
AFC	+0.08	0.48 (ns)	+0.11	0.37 (ns)

## Data Availability

The data presented in this study are available on request from the corresponding author. The data are not publicly available due to privacy and ethical restrictions involving patient-related information.

## Abbreviations

The following abbreviations are used in this manuscript:

AMH	Anti-Müllerian Hormone
FSH	Follicle-Stimulating Hormone
AFC	Antral Follicle Count
WHQ	Women’s Health Questionnaire
BRCA 1	Breast Cancer Gene 1
BRCA 2	Breast Cancer Gene 2
3D	Three-dimensional
H_0_	Null Hypothesis
H_1_	Alternative Hypothesis
X-ray	Röntgen Rays
ELISA	Enzyme-Linked Immunosorbent Assay
EIA	Enzyme Immunoassay
SonoAVC	Sonography-based Automated Volume Count
MRT	Mapi Research Trust
SPSS	Statistical Package for the Social Sciences
T1	Preoperative Score (WHQ)
T2	Postoperative Score (WHQ)
DEP	Depression (WHQ)
SOM	Somatic (WHQ)
ANX	Anxiety (WHQ)
SEX	Sexual (WHQ)
MEM	Memory (WHQ)
SLE	Sleep (WHQ)
VAS	Vasomotor (WHQ)
MEN	Menstrual (WHQ)
ATT	Attractiveness (WHQ)

## References

[1] Momenimovahed Z., Tiznobaik A., Taheri S., Salehiniya H. (2019). Ovarian Cancer in the World: Epidemiology and Risk Factors. Int. J. Womens Health.

[2] (2019). ACOG Committee Opinion No. 773 Summary: The Use of Antimüllerian Hormone in Women Not Seeking Fertility Care. Obstet. Gynecol..

[3] Daly M.B., Dresher C.W., Yates M.S., Jeter J.M., Karlan B.Y., Alberts D.S., Lu K.H. (2015). Salpingectomy as a Means to Reduce Ovarian Cancer Risk. Cancer Prev. Res..

[4] Cibula D., Widschwendter M., Majek O., Dusek L. (2011). Tubal Ligation and the Risk of Ovarian Cancer: Review and Meta-Analysis. Hum. Reprod. Update.

[5] Kurman R.J., Shih I.-M. (2011). Molecular Pathogenesis and Extraovarian Origin of Epithelial Ovarian Cancer—Shifting the Paradigm. Hum. Pathol..

[6] Yates M.S., Meyer L.A., Deavers M.T., Daniels M.S., Keeler E.R., Mok S.C., Gershenson D.M., Lu K.H. (2011). Microscopic and Early-Stage Ovarian Cancers in *BRCA1/2* Mutation Carriers: Building a Model for Early BRCA-Associated Tumorigenesis. Cancer Prev. Res..

[7] McAlpine J.N., Hanley G.E., Woo M.M.M., Tone A.A., Rozenberg N., Swenerton K.D., Gilks C.B., Finlayson S.J., Huntsman D.G., Miller D.M. (2014). Opportunistic Salpingectomy: Uptake, Risks, and Complications of a Regional Initiative for Ovarian Cancer Prevention. Am. J. Obstet. Gynecol..

[8] Backes F.J. (2014). Salpingectomy, Why Not?. Am. J. Obstet. Gynecol..

[9] Pölcher M., Wimberger P., Meinhold-Heerlein I., Runnebaum I., Schüler-Toprak S., Mahner S., Grimm C., Heinzelmann-Schwarz V., Hasenburg A., Sehouli J. (2025). Intergroup Statement: Opportunistic Salpingectomy—Molecular Pathology, Clinical Outcomes and Implications for Practice (German Ovarian Cancer Commission, the North-Eastern German Society of Gynecologic Oncology (NOGGO), AGO Austria and AGO Swiss). Arch. Gynecol. Obstet..

[10] Mills K., Marchand G., Sainz K., Azadi A., Ware K., Vallejo J., Anderson S., King A., Osborn A., Ruther S. (2021). Salpingectomy vs Tubal Ligation for Sterilization: A Systematic Review and Meta-Analysis. Am. J. Obstet. Gynecol..

[11] Zangmo R., Suresh G., Sarkar A., Ramu S., Roy K.K., Subramani K., Das P. (2024). The Effect of Salpingectomy on Ovarian Reserve Using Two Different Electrosurgical Instruments: Ultrasonic Shears Versus Bipolar Electrocautery. Cureus.

[12] Zadabedini Masouleh T., Etchegary H., Hodgkinson K., Wilson B.J., Dawson L. (2023). Beyond Sterilization: A Comprehensive Review on the Safety and Efficacy of Opportunistic Salpingectomy as a Preventative Strategy for Ovarian Cancer. Curr. Oncol..

[13] Nelson S.M., Davis S.R., Kalantaridou S., Lumsden M.A., Panay N., Anderson R.A. (2023). Anti-Müllerian Hormone for the Diagnosis and Prediction of Menopause: A Systematic Review. Hum. Reprod. Update.

[14] Kim C., Slaughter J.C., Wang E.T., Appiah D., Schreiner P., Leader B., Calderon-Margalit R., Sternfeld B., Siscovick D., Wellons M. (2017). Anti-Müllerian Hormone, Follicle Stimulating Hormone, Antral Follicle Count, and Risk of Menopause within 5 Years. Maturitas.

[15] Hunter M.S. (2003). The Women’s Health Questionnaire (WHQ): Frequently Asked Questions (FAQ). Health Qual. Life Outcomes.

[16] Moolhuijsen L.M.E., Visser J.A. (2020). Anti-Müllerian Hormone and Ovarian Reserve: Update on Assessing Ovarian Function. J. Clin. Endocrinol. Metab..

[17] Zhu Q., Li Y., Ma J., Ma H., Liang X. (2023). Potential Factors Result in Diminished Ovarian Reserve: A Comprehensive Review. J. Ovarian Res..

[18] Tavana Z., Askary E., Poordast T., Soltani M., Vaziri F. (2021). Does Laparoscopic Hysterectomy + Bilateral Salpingectomy Decrease the Ovarian Reserve More than Total Abdominal Hysterectomy? A Cohort Study, Measuring Anti-Müllerian Hormone before and after Surgery. BMC Womens Health.

[19] Vignarajan C.P., Malhotra N., Singh N. (2019). Ovarian Reserve and Assisted Reproductive Technique Outcomes After Laparoscopic Proximal Tubal Occlusion or Salpingectomy in Women with Hydrosalpinx Undergoing in Vitro Fertilization: A Randomized Controlled Trial. J. Minim. Invasive Gynecol..

[20] Ng K.Y.B., Cheong Y. (2019). Hydrosalpinx—Salpingostomy, Salpingectomy or Tubal Occlusion. Best Pract. Res. Clin. Obstet. Gynaecol..

[21] D’Arpe S., Franceschetti S., Caccetta J., Pietrangeli D., Muzii L., Panici P.B. (2015). Management of Hydrosalpinx before IVF: A Literature Review. J. Obstet. Gynaecol..

[22] Venturella R., Lico D., Borelli M., Imbrogno M.G., Cevenini G., Zupi E., Zullo F., Morelli M. (2017). 3 to 5 Years Later: Long-Term Effects of Prophylactic Bilateral Salpingectomy on Ovarian Function. J. Minim. Invasive Gynecol..

[23] Lashin H.A., El-Bakry M.A., El-sayed M.L.M., Gareeb M.A. (2021). Impact of Total Salpingectomy Versus Tubal Conservation During Abdominal Hysterectomy on Ovarian Function. Egypt. J. Hosp. Med..

[24] Wang S., Gu J. (2021). The Effect of Prophylactic Bilateral Salpingectomy on Ovarian Reserve in Patients Who Underwent Laparoscopic Hysterectomy. J. Ovarian Res..

[25] Rustamov O., Krishnan M., Roberts S.A., Fitzgerald C.T. (2016). Effect of Salpingectomy, Ovarian Cystectomy and Unilateral Salpingo-Oopherectomy on Ovarian Reserve. Gynecol. Surg..

[26] Farquhar C.M., Sadler L., Stewart A.W. (2008). A Prospective Study of Outcomes Five Years after Hysterectomy in Premenopausal Women. Aust. N. Z. J. Obstet. Gynaecol..

[27] Kuppermann M., Varner R.E., Summitt R.L., Learman L.A., Ireland C., Vittinghoff E., Stewart A.L., Lin F., Richter H.E., Showstack J. (2004). Effect of Hysterectomy vs Medical Treatment on Health-Related Quality of Life and Sexual Functioning: The Medicine or Surgery (Ms) Randomized Trial. JAMA.

[28] Freeman E.W., Sammel M.D., Lin H., Nelson D.B. (2006). Associations of Hormones and Menopausal Status with Depressed Mood in Women with No History of Depression. Arch. Gen. Psychiatry.

[29] Dennerstein L., Randolph J., Taffe J., Dudley E., Burger H. (2002). Hormones, Mood, Sexuality, and the Menopausal Transition. Fertil. Steril..

[30] Borud E.K., Martinussen M., Eggen A.E., Grimsgaard S. (2009). The Women’s Health Questionnaire (WHQ): A Psychometric Evaluation of the 36-item Norwegian Version. Scand. J. Psychol..

[31] Mohamed A.A., Yosef A.H., James C., Al-Hussaini T.K., Bedaiwy M.A., Amer S.A.K.S. (2017). Ovarian Reserve after Salpingectomy: A Systematic Review and Meta-analysis. Acta Obstet. Gynecol. Scand..

[32] Findley A.D., Siedhoff M.T., Hobbs K.A., Steege J.F., Carey E.T., McCall C.A., Steiner A.Z. (2013). Short-Term Effects of Salpingectomy during Laparoscopic Hysterectomy on Ovarian Reserve: A Pilot Randomized Controlled Trial. Fertil. Steril..

[33] Tehranian A., Zangbar R.H., Aghajani F., Sepidarkish M., Rafiei S., Esfidani T. (2017). Effects of Salpingectomy during Abdominal Hysterectomy on Ovarian Reserve: A Randomized Controlled Trial. Gynecol. Surg..

[34] Kotlyar A., Gingold J., Shue S., Falcone T. (2017). The Effect of Salpingectomy on Ovarian Function. J. Minim. Invasive Gynecol..

[35] Gelderblom M.E., IntHout J., Dagovic L., Hermens R.P.M.G., Piek J.M.J., De Hullu J.A. (2022). The Effect of Opportunistic Salpingectomy for Primary Prevention of Ovarian Cancer on Ovarian Reserve: A Systematic Review and Meta-Analysis. Maturitas.

[36] Ye X., Yang Y., Sun X. (2015). A Retrospective Analysis of the Effect of Salpingectomy on Serum antiMüllerian Hormone Level and Ovarian Reserve. Am. J. Obstet. Gynecol..

[37] Ely L.K., Truong M. (2017). The Role of Opportunistic Bilateral Salpingectomy vs Tubal Occlusion or Ligation for Ovarian Cancer Prophylaxis. J. Minim. Invasive Gynecol..

[38] Gu Y., Zhou G., Zhang M., Lu G., Shen F., Qi B., Hua K., Ding J. (2025). Bioengineered Extracellular Vesicles Presenting PD-L1 and Gal-9 to Ameliorate New-Onset Primary Ovarian Insufficiency (POI). Chem. Eng. J..

[39] Zhou G., Gu Y., Zhang M., Ding J., Lu G., Hua K., Shen F. (2025). Identification of Genetically Engineered Strategies to Manipulate Nano-Platforms Presenting Immunotherapeutic Ligands for Alleviating Primary Ovarian Insufficiency Progression. Cell Commun. Signal..

